# Robust Diagnosis Method Based on Parameter Estimation for an Interturn Short-Circuit Fault in Multipole PMSM under High-Speed Operation

**DOI:** 10.3390/s151129452

**Published:** 2015-11-20

**Authors:** Jewon Lee, Seokbae Moon, Hyeyun Jeong, Sang Woo Kim

**Affiliations:** Department of Electrical Engineering, Pohang University of Science and Technology (POSTECH), Room 304, LG Cooperative Electronics Engineering Building, 77 Cheongam-Ro, Nam-Gu, Pohang 37673, Korea; E-Mails: nowjjang@postech.ac.kr (J.L.); msbworld@postech.ac.kr (S.M.); jhy90@postech.ac.kr (H.J.)

**Keywords:** permanent magnet synchronous motor, interturn short, fault detection, diagnosis, fault index, parameter identification

## Abstract

This paper proposes a diagnosis method for a multipole permanent magnet synchronous motor (PMSM) under an interturn short circuit fault. Previous works in this area have suffered from the uncertainties of the PMSM parameters, which can lead to misdiagnosis. The proposed method estimates the q-axis inductance (*L_q_*) of the faulty PMSM to solve this problem. The proposed method also estimates the faulty phase and the value of *G*, which serves as an index of the severity of the fault. The *q*-axis current is used to estimate the faulty phase, the values of *G* and *L_q_*. For this reason, two open-loop observers and an optimization method based on a particle-swarm are implemented. The *q*-axis current of a healthy PMSM is estimated by the open-loop observer with the parameters of a healthy PMSM. The *L_q_* estimation significantly compensates for the estimation errors in high-speed operation. The experimental results demonstrate that the proposed method can estimate the faulty phase, *G*, and *L_q_* besides exhibiting robustness against parameter uncertainties.

## 1. Introduction

Motors have been used in many industrial applications, but faults in a motor system can cause system failure. Thus, fault diagnosis for motor systems is important and has been studied in recent years [1,2,3,4]. The permanent magnet synchronous motor (PMSM) exhibits various types of faults such as open-circuit/short-circuit faults [5,6], bearing fault [7], demagnetization fault [8] and eccentricity fault [9]. Short-circuit faults account for 21% of the faults in electrical machines, and most of them begin as interturn short-circuit faults [10], which occurs owing to degradation of the turn-to-turn insulation. This induces a high circulating current, and ohmic heat due to the current can degrade the wire insulation. As a result, the fault may become more serious.

Motor current signature analysis (MCSA) is a conventional fault detection method based on extraction of fault features. MCSA has commonly used harmonic components of stator currents as fault features [10,11]. MCSA is a non-invasive and efficient method; however, the harmonic components due to the fault are superimposed with those in a healthy PMSM [12]. The second-order harmonic component of the *q*-axis current (*i_q_*) was used to detect the interturn short-circuit fault, but the component had to be measured in advance for a healthy PMSM for various operations, because of the superposition [13].

Several fault diagnosis methods developed fault indices and used parameters of a healthy PMSM to estimate the fault indices. Stator currents in a healthy PMSM were calculated using the parameters of healthy PMSMs, and faults were detected from the differences between the calculated currents and the measured currents [6]. Aubert *et al.* and Grouz *et al.* estimated a ratio of shorted turns to total turns with fault models and the parameters [14,15]. The ratio is a fault-related parameter, and has a constant value for any operational condition. As a result, those methods were rarely affected by the operational condition of the PMSM. Utilization of the parameters of a healthy PMSM is an efficient methodology, because premeasurements for various operations are no longer necessary. The parameter values are conventionally assumed as the known values. However, they can change. Therefore, the parameter values used in fault diagnosis can be different from the true values, and the difference can lead to misdiagnosis. For a healthy PMSM, these parameters can be estimated by the online parameter estimation method [16]. However, the fault affects the estimation [17], and therefore, the conventional method cannot be applied without modification.

We propose a novel diagnosis method with robustness against the parameter uncertainties. This method estimates the fault phase vector (***p***), a fault-related parameter (*G*), and the *q*-axis inductance (*L_q_*). The novelty of this fault diagnosis lies in the *L_q_* estimation method for PMSM under the interturn short-circuit fault. *G* is used as an index of the severity of the fault and is calculated from the stator resistance, the fault resistance, and the ratio of shorted turns to total turns [18]. In the proposed method, the *q*-axis current (*i_q_*) is measured, and the *q*-axis current of a healthy PMSM (*i_q,n_*) is estimated using the known parameters of a healthy PMSM, ***p*** and *G* are estimated from *i_q_* − *i_q,n_*. However, the inaccuracy of the known parameters results in a significant error in *i_q,n_* estimation, which, in turn, leads to a significant error in *G* estimation. In high-speed operation, *L_q_* is the primary parameter used in the calculation of *i_q,n_*, and, therefore, *L_q_* estimation significantly compensates for the estimation errors. As a result, the proposed method exhibits robustness against the inaccuracy of the known parameters.

This paper is organized as follows. Section 2 describes a mathematical model for a multipole PMSM under an interturn short-circuit fault. Section 3 describes the proposed fault diagnosis method. The proposed method is verified by experiment results in Section 4. Finally, concluding remarks are made in Section 5.

## 2. Mathematical Model for Multipole PMSM under Interturn Short Circuit Fault

**Figure 1:**
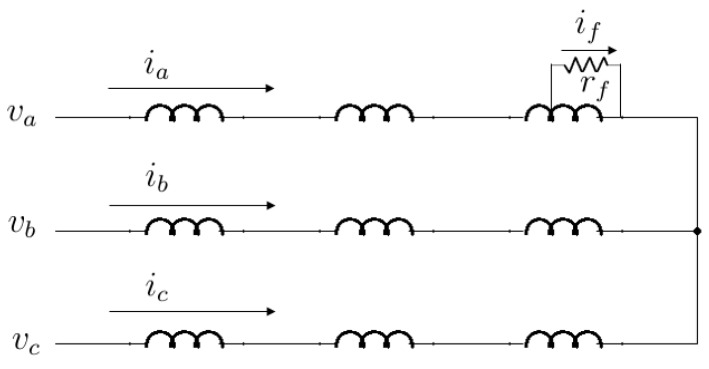
An interturn short-circuit fault in phase *a* of stator windings.

Figure 1 shows serial-connected windings in a multipole PMSM under an interturn short-circuit fault. A fault resistance *R_f_* denotes the insulation degradation between turns of a winding, and *μ* is the ratio of short-circuited turns to the total turns of the faulty winding. This additional loop circuit causes a circulating current *i_f_*.

Gu *et al.* developed an interturn short-circuit fault model for a multipole PMSM [19]. The fault model can be modified as

(1) $$\begin{array}{c} v_{abc}=R_{s}i_{abc}+\frac{d}{dt}\left[Li_{abc}+\lambda_{PM}\right]-R_{s}pK\mu i_{f}-\frac{d}{dt}LpK\mu i_{f} \end{array}$$

where

$$\begin{array}{c} v_{abc}=\left[\begin{array}{c} v_{a}\\ v_{b}\\ v_{c} \end{array}\right],i_{abc}=\left[\begin{array}{c} i_{a}\\ i_{b}\\ i_{c} \end{array}\right],\lambda_{PM}=\psi_{pm}\left[\begin{array}{c} \cos(\theta )\\ \cos(\theta -\frac{2\pi }{3})\\ \cos(\theta +\frac{2\pi }{3}) \end{array}\right],L=\left[\begin{array}{ccc} L_{a} & M_{ab} & M_{ac}\\ M_{ab} & L_{b} & M_{bc}\\ M_{ac} & M_{bc} & L_{c} \end{array}\right] \end{array}$$

*K* is a coefficient determined by the structure of the winding connections (*K* = 1/*N* for a serial-connected-winding PMSM and *K* = 1 for a parallel-connected-winding PMSM), *N* is the number of windings per phase, and ***p*** is a faulty phase vector. If an interturn short-circuit fault occurs in phase *a*, *b* or *c*, then ***p*** = [1 0 0]^*T*^, [0 1 0]^*T*^, or [0 0 1]^*T*^. This paper uses the conventional angular-position-dependent inductances for an interior PMSM (IPMSM)

(2) $$\begin{array}{cc} L= & \left[\begin{array}{ccc} L_{ms}+L_{l} & -\frac{1}{2}L_{ms} & -\frac{1}{2}L_{ms}\\ -\frac{1}{2}L_{ms} & L_{ms}+L_{l} & -\frac{1}{2}L_{ms}\\ -\frac{1}{2}L_{ms} & -\frac{1}{2}L_{ms} & L_{ms}+L_{l} \end{array}\right]-L_{\delta }\left[\begin{array}{ccc} \cos2\theta & \cos(2\theta -\frac{2\pi }{3}) & \cos(2\theta +\frac{2\pi }{3})\\ \cos(2\theta -\frac{2\pi }{3}) & \cos(2\theta +\frac{2\pi }{3}) & \cos2\theta \\ \cos(2\theta +\frac{2\pi }{3}) & \cos2\theta & \cos(2\theta -\frac{2\pi }{3}) \end{array}\right] \end{array}$$

where *θ* is the electrical angular position of the IPMSM [20].

The other model equation is determined by the structure of the winding connections. Equation (3) is for the parallel winding connections and Equation (4) is for the serial winding connections.

(3) $$\begin{array}{cc} \frac{R_{f}i_{f}}{\mu }= & p^{T}v_{abc}-\left(1-\mu \right)NR_{s}i_{f} \end{array}$$

(4) $$\begin{array}{cc} \frac{R_{f}i_{f}N}{\mu }= & p^{T}v_{abc}-\left(1-\frac{\mu }{N}\right)R_{s}i_{f}-\frac{d}{dt}\left[\left(\frac{1}{1-\gamma }-\frac{1}{N}\right)L_{m}+\left(1-\frac{1}{N}\right)L_{l}\right]\mu i_{f} \end{array}$$

where *γ* is a flux coupling coefficient between the stator windings in the same phase, and *L_m_* and *L_l_* represent the mutual and leakage inductance components of the self-inductance in the faulty phase, respectively. Equations (3) and (4) can be rearranged as follows:

(5) $$\begin{array}{cc} p^{T}v_{abc}= & \left[\frac{R_{f}}{\mu^{2}}+\left(\frac{1}{\mu }-1\right)NR_{s}\right]K\mu i_{f} \end{array}$$

(6) $$\begin{array}{cc} p^{T}v_{abc}= & \left[\frac{R_{f}N^{2}}{\mu^{2}}+\left(\frac{N}{\mu }-1\right)R_{s}\right]K\mu i_{f}+\frac{d}{dt}\left[\left(\frac{N}{1-\gamma }-1\right)L_{m}+\left(N-1\right)L_{l}\right]K\mu i_{f} \end{array}$$

**Figure 2:**
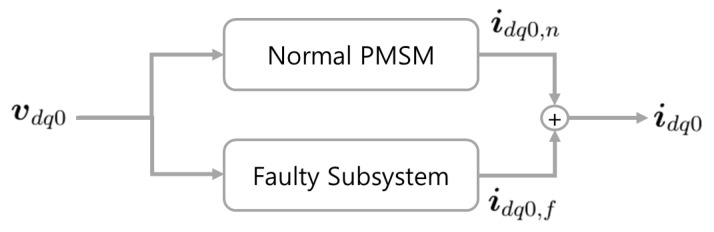
Two subsystems of PMSM under interturn short-circuit fault.

Equation (1) can be transformed by Park’s transformation matrix *T_park_* [14,21].

(7) $$\begin{array}{c} v_{dq0}=\left[\begin{array}{ccc} L_{d} & 0 & 0\\ 0 & L_{q} & 0\\ 0 & 0 & L_{l} \end{array}\right]\frac{d}{dt}\left(i_{dq0}-i_{dq0,f}\right)+\left[\begin{array}{ccc} R_{s} & -\omega L_{q} & 0\\ \omega L_{d} & R_{s} & 0\\ 0 & 0 & R_{s} \end{array}\right]\left(i_{dq0}-i_{dq0,f}\right)+\left[\begin{array}{c} 0\\ \omega \psi_{PM}\\ 0 \end{array}\right] \end{array}$$

where *ω* is the electrical angular velocity of the PMSM, and ***i***_*dq*0,*f*_ = ***T**_park_* × *pKμi_f_*. Let ***i***_*dq*0,*n*_ be defined as ***i***_*dq*0_ − ***i***_*dq*0,*f*_. Then, Equation (7) is the same as the model equation for a normal PMSM with stator currents ***i***_*dq*0,*n*_. When the stator voltages are given, ***i***_*dq*0,*f*_ is uniquely determined from Equation (5) or (6) with ***T**_park_*, and also ***i***_*dq*0,*n*_ from Equation (7). This implies that ***i***_*dq*0,*n*_ and ***i***_*dq*0,*f*_ can be considered as states that are independent of each other, and therefore, the faulty PMSM system can be divided into two subsystems, as shown in Figure 2. The "Faulty Subsystem" is described by Equation (5) or (6), and the “Normal PMSM” by Equation (7).

## 3. The Proposed Fault Detection Method for Interturn Short-Circuit Fault

In the development of the proposed method, the following accents for letters will be used. “ ^ ” denotes the estimated value, and “ ˜ ” denotes the estimation error. “ ′ ” denotes the known value of a parameter before estimation, and “Δ” denotes the uncertainty of the parameter, which is the difference between the known value and the true value of a parameter; for example, $\Delta L_{d}=L_{d}^{\prime }-L_{d}$. Non-accented letters denote true values.

Let *G* denote the reciprocal of $\frac{R_{f}}{\mu^{2}}+\left(\frac{1}{\mu }-1\right)NR_{s}$ in Equation (5) and $\frac{R_{f}N^{2}}{\mu^{2}}+\left(\frac{N}{\mu }-1\right)R_{s}$ in Equation (6). Then, it can be used as the fault index, because *G* comprises the fault-related parameters (*μ*, *R_f_*) and is constant value if *R_f_*, *μ*, *R_s_* are not changed [18]. In addition, *μ*, which is used as the fault index in other studies [14,15], can be calculated from *G* under the assumption that *R_f_* = 0. Therefore, *G* is used as the fault index in the proposed method. The faulty phase can be determined from ***p***.

**Figure 3:**
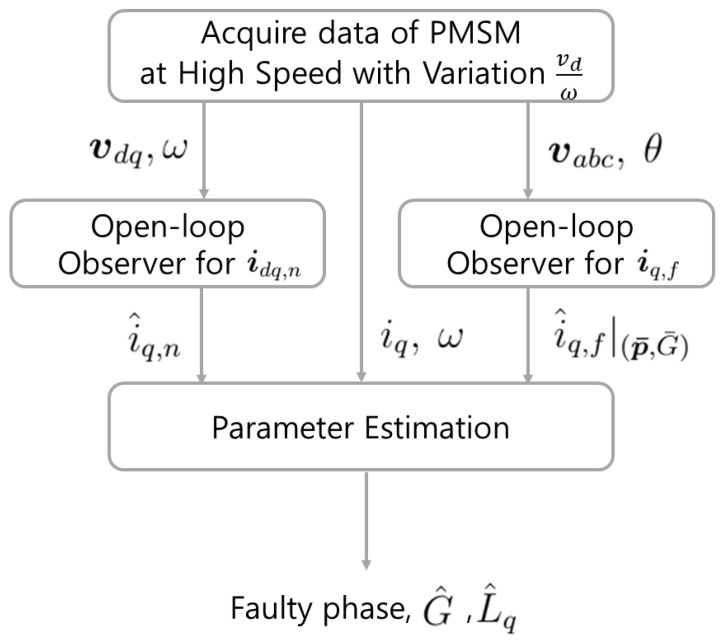
Diagram of proposed method.

The objective of the proposed method is the estimation of ***p*** and *G*. Figure 3 shows the diagram of the proposed method. First, the stator voltage, current, angular velocity, and position data are acquired when the PMSM runs at high speed with $\frac{v_{d}}{\omega }$ variation.

Two open-loop observers are implemented for the parameter estimation. *i_q,f_* and *i_q,n_* have to be estimated by the open-loop observers because they cannot be measured. The open-loop observer for ***i**_dq,n_* estimates *i_q,n_* with the known PMSM parameters $\left(L_{d}^{\prime },L_{q}^{\prime },R_{s}^{\prime },\psi_{PM}^{\prime }\right)$. The uncertainties of the parameters result in an estimation error of *i_q,n_*, and the most significant error is caused by Δ*L_q_* in the high-speed operation. For this reason, *L_q_* is also estimated in the parameter estimation, and, consequently, the error caused by Δ*L_q_* will be compensated. The open-loop observer for *i_q,f_* estimates many $i_{q,f}{|}_{\left(\bar{p},\bar{G}\right)}$ values for the candidates ($\bar{p},\bar{G}$).

The parameter estimation process estimates ***p***, *G* and *L_q_*. ***p*** and *G* can be uniquely determined from *i_q,f_*. However, *i_q,f_* cannot be measured and or even directly estimated. Instead, $i_{q}-{\hat{i}}_{q,n}$ is used because *i_q_* − *i_q,n_* is equivalent to *i_q,f_*. Optimization based on a particle-swarm is implemented to estimate the faulty parameters as in [15]. Among ${\hat{i}}_{q,f}{|}_{\left(\bar{p},\bar{G}\right)}$ for candidates ($\bar{p},\bar{G}$), the optimization finds the closest ${\hat{i}}_{q,f}{|}_{\left(\bar{p},\bar{G}\right)}$ to *i_q_* − *i_q,n_* in the least-squares sense. The $\bar{p},\bar{G}$ of the result are denoted as $\hat{p},\hat{G}$.

After the parameter estimation, the open-loop observer for $i_{q,f}$ recalculates ${\hat{i}}_{q,f}{|}_{\left(\bar{p},\bar{G}\right)}$ with ${\hat{L}}_{q}$ for a more precise estimation, and then $\left(\hat{p},\hat{G},{\hat{L}}_{q}\right)$ are reestimated in the parameter estimation as the final result. Finally, the faulty phase is determined from $\hat{p}$. The following subsections describe the specifications of the processes.

### 3.1. Open-Loop Observer for i_q,f_

${\hat{i}}_{q,f}{|}_{\left(\bar{p},\bar{G}\right)}$ is calculated from $T_{park}\times \bar{p}K{\hat{\mu i}}_{f}{|}_{\left(\bar{p},\bar{G}\right)}$. $K\hat{\mu i_{f}}$ can be calculated from Equation (5) or (6) as follows:

(8) $$\begin{array}{cc} K{\hat{\mu i}}_{f}{|}_{\left(\bar{p},\bar{G}\right)}\left(m\right) & =\bar{G}{\bar{p}}^{T}v_{abc}\left(m\right) \end{array}$$

(9) $$\begin{array}{cc} K{\hat{\mu i}}_{f}{|}_{\left(\bar{p},\bar{G}\right)}\left(m+1\right) & =e^{-\frac{T_{s}}{L\left(m\right)\bar{G}}}\times K{\hat{\mu i}}_{f}{|}_{\left(\bar{p},\bar{G}\right)}\left(m\right)+\left(1-e^{-\frac{T_{s}}{L\left(m\right)\bar{G}}}\right)\bar{G}{\bar{p}}^{T}v_{abc}\left(m\right) \end{array}$$

where *L*(*m*) is $\left(\frac{N}{1-\gamma }-1\right)L_{m}+\left(N-1\right)L_{l}$ at sample time *m*. *L_m_* can be calculated with $\bar{p},L_{d},L_{q},$ and *L_l_* [20,22],

(10) $$\begin{array}{c} L_{m}=\frac{L_{d}+L_{q}-2L_{l}}{3}-\frac{\left(L_{q}-L_{d}\right)}{3}\cos\left(2\theta +\phi_{\bar{p}}\right) \end{array}$$

where $\phi_{\bar{p}}=0,\frac{2\pi }{3},-\frac{2\pi }{3}$ for $\bar{p}={\left[1\;0\;0\right]}^{T},{\left[0\;1\;0\right]}^{T},{\left[0\;0\;1\right]}^{T}$, respectively. In a real-world application, $L_{d}^{\prime },L_{q}^{\prime }$ and $L_{l}^{\prime }$ are used instead.

$K{\hat{\mu i}}_{f}{|}_{\left(\bar{p},\bar{G}\right)}$ in Equation (8) does not depend on its own previous value and the PMSM parameters, and is therefore calculated without an estimation error for a given $\bar{p}$ and $\bar{G}$. On the other hand, in Equation (9), it has the dependencies and the estimation error of $K\mu i_{f}{|}_{\left(\bar{p},\bar{G}\right)}$. However, for low values of $\bar{G}$, $-\frac{T_{s}}{L\left(m\right)\bar{G}}$ is a large negative number, and $K{\hat{\mu i}}_{f}{|}_{\left(\bar{p},\bar{G}\right)}\left(m\right)$ is almost identical to $\bar{G}{\bar{p}}^{T}v_{abc}\left(m\right)$, making the estimation error negligible.

### 3.2. Open-Loop Observer for **i**_dq,n_

Equation (7) can be easily expressed as a state equation for ***i**_dq,n_*. With a sufficiently high sample rate, the estimation value of ***i**_dq,n_* for *m* + 1 samples can be calculated by

(11) $$\begin{array}{c} \left[\begin{array}{c} {\hat{i}}_{d,n}\left(m+1\right)\\ {\hat{i}}_{q,n}\left(m+1\right) \end{array}\right]=e^{AT_{s}}\left[\begin{array}{c} {\hat{i}}_{d,n}\left(m\right)\\ {\hat{i}}_{q,n}\left(m\right) \end{array}\right]-\left(I_{2}-e^{AT_{s}}\right)A^{-1}\left[\begin{array}{c} \frac{v_{d}\left(m\right)}{L_{d}}\\ \frac{v_{q}\left(m\right)-\omega \left(m\right)\psi_{PM}}{L_{q}} \end{array}\right] \end{array}$$

where $A=\left[\begin{array}{cc} -\frac{R_{s}}{L_{d}} & \frac{\omega \left(m\right)L_{q}}{L_{d}}\\ -\frac{\omega \left(m\right)L_{d}}{L_{q}} & -\frac{R_{s}}{L_{q}} \end{array}\right],I_{2}=\left[\begin{array}{cc} 1 & 0\\ 0 & 1 \end{array}\right],T_{s}$ is the sampling period. The matrix ***A*** has two eigenvalues given by $-\left(\frac{1}{L_{d}}+\frac{1}{L_{q}}\right)\frac{R_{s}}{2}\pm \sqrt{{\left(\frac{1}{L_{d}}-\frac{1}{L_{q}}\right)}^{2}\frac{R_{s}^{2}}{4}-\omega^{2}}$. The upper bound of the real parts is $-\frac{R_{s}}{L_{q}}$ if *L_q_* ≥ *L_d_*. Because *L_q_* is usually much smaller than *R_s_*, the real parts are large negative numbers. This means that $e^{AT_{s}}$ rapidly decreases with time, and thus the initial estimation error diminishes after sufficient time elapses. In addition, this observer has a high convergence rate to the steady state.

However, as mentioned earlier, the open-loop observer suffers from the critical problem that the estimation error ${\tilde{i}}_{dq,n}$ is greatly affected by parameter uncertainties. Because of the high convergence rate, only the estimation error in the steady state is taken into consideration. Using $e^{AT_{s}}\approx 0$, Equation (11) can be modified as follows:

(12) $$\begin{array}{c} \left[\begin{array}{c} {\hat{i}}_{d,n}\\ {\hat{i}}_{q,n} \end{array}\right]=-A^{-1}\left[\begin{array}{c} \frac{v_{d}}{L_{d}}\\ \frac{v_{q}-\omega \psi_{PM}}{L_{q}} \end{array}\right]=\frac{1}{R_{s}^{2}+\omega^{2}L_{d}L_{q}}\left[\begin{array}{c} R_{s}v_{d}+\omega L_{q}\left(v_{q}-\omega \psi_{PM}\right)\\ -\omega L_{d}v_{d}+R_{s}\left(v_{q}-\omega \psi_{PM}\right) \end{array}\right] \end{array}$$

For the high-speed operation, $R_{s}^{2}+\omega^{2}L_{d}L_{q}\approx \omega^{2}L_{d}L_{q}$. If the observer uses $L_{q}^{\prime }$ instead of *L_q_*, the estimation error can be calculated as

(13) $$\begin{array}{c} \left[\begin{array}{c} \frac{R_{s}}{\omega L_{d}\left(L_{q}+\Delta L_{q}\right)}\frac{v_{d}}{\omega }+\frac{1}{L_{d}}\left(\frac{v_{q}}{\omega }-\psi_{PM}\right)\\ -\frac{1}{\left(L_{q}+\Delta L_{q}\right)}\frac{v_{d}}{\omega }+\frac{R_{s}}{\omega L_{d}\left(L_{q}+\Delta L_{q}\right)}\left(\frac{v_{q}}{\omega }-\psi_{PM}\right) \end{array}\right]-\left[\begin{array}{c} \frac{R_{s}}{\omega L_{d}L_{q}}\frac{v_{d}}{\omega }+\frac{1}{L_{d}}\left(\frac{v_{q}}{\omega }-\psi_{PM}\right)\\ -\frac{1}{L_{q}}\frac{v_{d}}{\omega }+\frac{R_{s}}{\omega L_{d}L_{q}}\left(\frac{v_{q}}{\omega }-\psi_{PM}\right) \end{array}\right]\\ =\left[\begin{array}{c} -\frac{R_{s}}{\omega L_{d}}\frac{v_{d}}{\omega }\\ \frac{v_{d}}{\omega }-\frac{R_{s}}{\omega L_{d}}\left(\frac{v_{q}}{\omega }-\psi_{PM}\right) \end{array}\right]\frac{\Delta L_{q}}{L_{q}\left(L_{q}+\Delta L_{q}\right)}=\left[\begin{array}{c} -\frac{R_{s}}{\omega L_{d}}\frac{v_{d}}{\omega }\\ \frac{v_{d}}{\omega }-\frac{R_{s}}{\omega L_{d}}\left(\frac{v_{q}}{\omega }-\psi_{PM}\right) \end{array}\right]\frac{\alpha }{L_{q}+\Delta L_{q}} \end{array}$$

where *α* = Δ*L_q_*/*L_q_*. In the same way, the estimation error under the other parameter uncertainties can be described as

(14) $$\begin{array}{cc} & \left[\begin{array}{c} -\frac{R_{s}}{\omega L_{q}}\frac{v_{d}}{\omega }-\left(\frac{v_{q}}{\omega }-\psi_{PM}\right)\\ -\frac{R_{s}}{\omega L_{q}}\left(\frac{v_{q}}{\omega }-\psi_{PM}\right) \end{array}\right]\frac{\beta }{L_{d}^{\prime }}\;\;\;\;\;\;\;\;\;\;\;\;\;\;\;\;\;\;\;\;\;\;\;\text{with}\;\;L_{d}^{\prime } \end{array}$$

(15) $$\begin{array}{cc} & \left[\begin{array}{c} -\frac{1}{L_{d}}\\ -\frac{R_{s}}{\omega L_{d}L_{q}} \end{array}\right]\Delta \psi_{PM}\;\;\;\;\;\;\;\;\;\;\;\;\;\;\;\;\;\;\;\;\;\;\;\;\;\;\;\;\;\;\;\;\;\;\;\;\;\;\;\;\;\;\text{with}\;\;\psi_{PM}^{\prime } \end{array}$$

(16) $$\begin{array}{cc} & \left[\begin{array}{c} \frac{1}{\omega L_{d}L_{q}}\frac{v_{d}}{\omega }\\ \frac{1}{\omega L_{d}L_{q}}\left(\frac{v_{q}}{\omega }-\psi_{PM}\right) \end{array}\right]\Delta R_{s}\;\;\;\;\;\;\;\;\;\;\;\;\;\;\;\;\;\;\;\;\;\;\;\;\;\;\;\;\;\;\text{with}\;\;R_{s}^{\prime } \end{array}$$

where *β* = Δ*L_d_*/*L_d_*. In conclusion, the estimation error of the observer with uncertain parameters can be approximated as the sum of Equations (13)–(16)

(17) $$\begin{array}{c} \left[\begin{array}{c} {\tilde{i}}_{d,n}\\ {\tilde{i}}_{q,n} \end{array}\right]\approx \left[\begin{array}{c} -\frac{R_{s}}{\omega L_{d}}\frac{v_{d}}{\omega }\\ \frac{v_{d}}{\omega }-\frac{R_{s}}{\omega L_{d}}\left(\frac{v_{q}}{\omega }-\psi_{PM}\right) \end{array}\right]\frac{\alpha }{L_{q}^{\prime }}+\left[\begin{array}{c} -\frac{R_{s}}{\omega L_{q}}\frac{v_{d}}{\omega }-\left(\frac{v_{q}}{\omega }-\psi_{PM}\right)\\ -\frac{R_{s}}{\omega L_{q}}\left(\frac{v_{q}}{\omega }-\psi_{PM}\right) \end{array}\right]\frac{\beta }{L_{d}^{\prime }}\\ +\left[\begin{array}{c} -\frac{1}{L_{d}}\\ -\frac{R_{s}}{\omega L_{d}L_{q}} \end{array}\right]\Delta \psi_{PM}+\left[\begin{array}{c} \frac{R_{s}}{\omega L_{d}L_{q}}\frac{v_{d}}{\omega }\\ \frac{R_{s}}{\omega L_{d}L_{q}}\left(\frac{v_{q}}{\omega }-\psi_{PM}\right) \end{array}\right]\frac{\Delta R_{s}}{R_{s}} \end{array}$$

### 3.3. Parameter Estimation

This process estimates ***p***, *G* and *L_q_* in the least-squares sense. Because *i_q,f_* = *i_q_* − *i_q,n_*, (***p***, *G*) equals the optimal solution, which minimizes the cost function $\sum {\left(i_{q}-i_{q,n}-i_{q,f}{|}_{\left(\bar{p},\bar{G}\right)}\right)}^{2}$. ${\tilde{i}}_{q,n}$ from Equation (17) is added in the cost function to compensate the estimation error of ${\hat{i}}_{q,n}$. Therefore, the following problem can be used for parameter estimation:

(18) $$\begin{array}{c} \text{argmin}\;\sum_{m}{\left[i_{q}\left(m\right)-\left({\hat{i}}_{q,n}\left(m\right)-{\tilde{i}}_{q,n}\left(m\right)\right)-{\hat{i}}_{q,f}{|}_{\left(\bar{p},\bar{G}\right)}\left(m\right)\right]}^{2} \end{array}$$

***p***, *G* and the uncertain parameters in Equation (17) are identified from the solutions.

However, the estimation for all the parameters requires high computational complexity; therefore, the number of estimated parameters should be reduced. In high-speed operation, the voltage drops over the stator resistance are relatively small [20]; therefore, *R_s_*-related terms have small values. On the other hand, $\frac{v_{d}}{\omega }\frac{\alpha }{L_{q}^{\prime }}$ is the most significant term. For example, in the experiment, the PMSM had run at its rated speed and torque, *ω* = 1413 rad/s, $\frac{v_{d}}{\omega }=-0.045$ V· s/rad, and $\frac{v_{q}}{\omega }-\psi_{PM}=-0.0125$ V· s/rad. If 10% higher parameter values are used in this observer, then Equation (17) becomes

(19) $$\begin{array}{c} \left[\begin{array}{c} {\tilde{i}}_{d,n}\\ {\tilde{i}}_{q,n} \end{array}\right]\approx \left[\begin{array}{c} 0.076\\ -0.748+0.021 \end{array}\right]+\left[\begin{array}{c} 0.456\\ 0.021 \end{array}\right]+\left[\begin{array}{c} -3.156\\ 0.179 \end{array}\right]+\left[\begin{array}{c} 0.082\\ 0.023 \end{array}\right]=\left[\begin{array}{c} -2.542\\ -0.504 \end{array}\right] \end{array}$$

Thus, ${\tilde{i}}_{q,n}$ can be significantly reduced by estimation for *α* in Equation (17).

Finally, the following problem will be solved in the proposed method for a PMSM under high-speed operation.

(20) $$\begin{array}{c} \underset{\bar{p},\bar{G},\bar{\alpha }}{\text{argmin}}\;\sum_{m}{\left[i_{q}\left(m\right)-\left({\hat{i}}_{q,n}\left(m\right)-\frac{v_{d}\left(m\right)}{\omega (m)}\frac{\bar{\alpha }}{L_{q}^{\prime }}\right)-{\hat{i}}_{q,f}{|}_{\left(\bar{p},\bar{G}\right)}\left(m\right)\right]}^{2} \end{array}$$

The variation in $\frac{v_{d}}{\omega }$ is necessary to estimate *α*. Let $\hat{p},\hat{G},\hat{\alpha },{\hat{L}}_{q}$ denote the optimal solutions of the preceding problem. ${\hat{L}}_{q}$ is estimated by $L_{q}^{\prime }/\left(1+\hat{\alpha }\right)$. To solve this problem, uniformly-distributed-points of $\left(\bar{\alpha },\bar{G}\right)$ (a particle-swarm) are generated for each $\bar{p}$, and the minimum point for Equation (20) is found. The following section demonstrates the validity of the proposed method.

## 4. Experiment and Discussion

**Figure 4:**
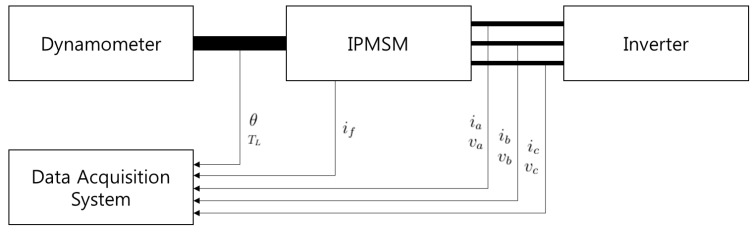
The experimental platform.

Figure 4 shows the experimental platform. A model FMAIN22-BBFB1 IPMSM manufactured by Higen Motor was used in the experiments. Table 1 shows the specifications of the IPMSM. The motor has three phases with wye connection, and five branches per phase. Each branch consists of 6(=*N*) concentrated windings with a serial connection, and its resistance is 1.85 ohm. Each winding has 20 turns. The IPMSM was driven by a LSIS SV022iG5A-2 inverter and loaded with a Magtrol HD-715 hysteresis dynamometer, which generated an accurate load torque and measured the output torque of the motor (*T_L_*). Stator currents and voltages, the circulating current, the angular position, and the output torque were measured with the sample frequency of 100 kHz. Low-pass filtering with a cutoff frequency 1 kHz was adopted to eliminate the frequencies from the inverter switching.

**Table 1:** Specifications of interior permanent magnet synchronous motor (IPMSM) used in experiment.

Parameter	Value
Rated output	2.2 kW
Number of pole pairs	3
Rated current	8.2 A
Rated RPM	4500 rpm
Rated torque	4.7 N·m
Input voltage	212 V
*d*-axis inductance *L_d_*	3.09 mH
*q*-axis inductance *L_q_*	5.47 mH
Leakage inductance *L_l_*	0.60 mH
Stator resistance *R_s_*	0.44 ohm
Magnet flux linkage *ψ_PM_*	0.0976 Wb

**Figure 5:**
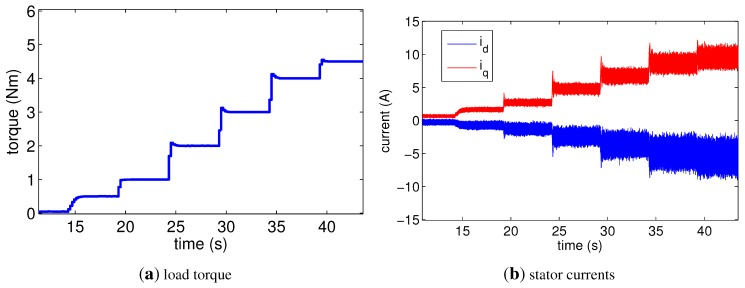
(**a**) Plan of load torque used in the experiment; (**b**) Stator currents in healthy motor under the plan.

The experiments were conducted on a healthy motor and a faulty motor. The interturn short-circuit fault was realized by connecting a resistor between the wire turns of the first winding in the first branch of phase *c*, in other words, ***p*** = [0 0 1]^*T*^. Five resistors of *R_f_* = [0.22, 0.36, 0.60, 1.12, 2.14 Ω] and the three ratios *μ* = [5/20, 10/20, 15/20] were used to implement the various severity levels of the fault. As a result, a total of 15 levels of severity in the interturn short-circuit fault were achieved. Table 2 shows the values of *G* calculated from $\frac{1}{G}=\frac{R_{f}N^{2}}{\mu^{2}}+\left(\frac{N}{\mu }-1\right)R_{s}$, but the resistance of the branch was instead used as *R_s_*. This modification was necessary because the structure did not completely consist of serial-winding-connections. These values are considered true values and are used on the *x*-axis in the plots. In addition, the G value of the healthy motor was zero. For high-speed operation under a variation of $\frac{v_{d}}{\omega }$, both motors ran at 3000, 4000 and 4500 rpm under increasing load torque, as shown in Figure 5a. Under the load torque, the stator currents in the healthy motor were driven as shown in Figure 5b, and $\frac{v_{d}}{\omega }$ decreased from 0 to −0.06 V·s/rad. In the experiments, the *γ* values of the two motors were unknown. However, it was known that they were small values, and thus, *γ* ≈ 0 was used in the proposed method. Further, $L_{l}^{\prime }=0.6$ mH and $R_{s}^{\prime }=0.44$ Ω were given.

**Table 2:** *G* values of the realized faults in the faulty motor.

*G* (mΩ^−1^)					
*μ*	*R_f_* (Ω)				
	0.22	0.36	0.60	1.12	2.14
5/20	5.9	4.0	2.6	1.5	0.8
10/20	19.2	13.9	9.4	5.5	3.0
15/20	37.0	27.8	19.5	11.8	6.7

### 4.1. Estimation Results of Observers and Parameter Estimation

**Figure 6:**
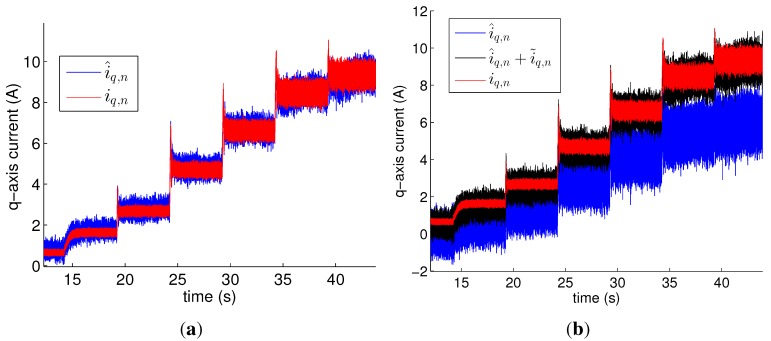
Measured values of *q*-axis current (*i_q,n_*) in healthy motor and estimated values (${\hat{i}}_{q,n}$) obtained by open-loop observer for *i_q,n_* using (**a**) accurate values of parameters; (**b**) wrong values such as $L_{q}^{\prime }=8.0$ mH, $L_{d}^{\prime }=4.0$ mH, and $\psi_{PM}^{\prime }=0.1464$ Wb. The healthy motor ran at 4500 rpm.

Figure 6 depicts the estimation results obtained by the open-loop observer for *i_q,n_* for the healthy motor. As shown in Figure 6a, the plot for the estimated values was close to that for the measured values when the observer had used the accurate parameters in Table 1. Their DC components were almost equal, but a difference in the AC components appeared and varied within ±1 A. This estimation error cannot be eliminated because the faulty model has uncertainty. Figure 6b shows that ${\hat{i}}_{q,n}$ had significant estimation errors when the observer used wrong values such as $L_{q}^{\prime }=8.0$ mH, $L_{d}^{\prime }=4.0$ mH, and $\psi_{PM}^{\prime }=0.1464$ Wb. However, the errors were significantly reduced as ${\tilde{i}}_{q,n}$ in Equation (17) was added to ${\hat{i}}_{q,n}$.

**Figure 7:**
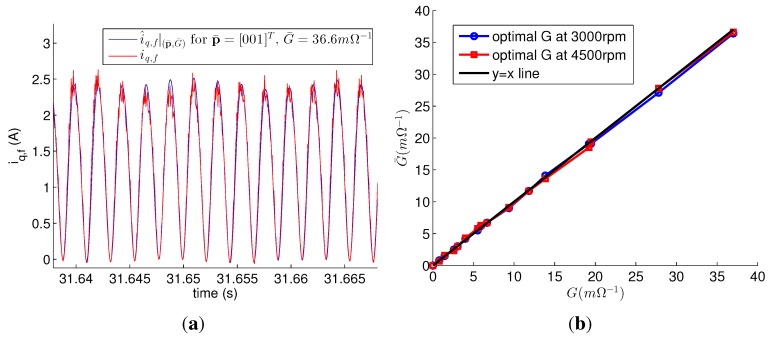
(**a**) ${\hat{i}}_{q,f}{|}_{\bar{p},\bar{G}}$ for $\bar{p}={\left[0\;0\;1\right]}^{T}$, $\bar{G}=36.6$ mΩ^−1^, and the measurement data *i_q,f_* of faulty motor under the fault ***p*** = [0 0 1]^*T*^, *G* = 37.0 mΩ^−1^; (**b**) Optimal $\bar{G}$ for the different fault severities and speeds.

Figure 7a shows *i_q,f_* and ${\hat{i}}_{q,f}{|}_{\left(\bar{p},\bar{G}\right)}$ for $\bar{p}={\left[0\;0\;1\right]}^{T}$ and $\bar{G}=36.6$ mΩ^−1^ obtained by the observer for *i_q,f_* with accurate values of the parameters. *i_q,f_* was calculated from its definition with measured data of *i_f_* in the faulty motor. The faulty condition was *G* = 37.0 mΩ^−1^ (*R_f_* = 0.22 Ω, *μ* = 15/20). ${\hat{i}}_{q,f}{|}_{\left(\bar{p},\bar{G}\right)}$ for $\bar{p}={\left[0\;0\;1\right]}^{T},\bar{G}=36.6$ mΩ^−1^ was the optimal solution to minimize $\sum {\left[i_{q,f}-{\hat{i}}_{q,f}{|}_{\left(\bar{p},\bar{G}\right)}\right]}^{2}$, and was almost the same as *i_q,f_*. Figure 7b shows the values of the optimal solution for different fault severities and speeds. Every optimal $\bar{G}$ almost equals *G*. In addition, the faulty phases according to the optimal $\bar{p}$ were all phase *c* in both speed, except for the healthy motor.

In conclusion, both observers with accurate parameters estimated *i_q,n_* and *i_q,f_* with good accuracy, and ${\tilde{i}}_{q,n}$ from Equation (17) was valid. In addition, ***p*** and *G* were uniquely determined from *i_q,f_* by solving $\underset{\left(\bar{p},\bar{G}\right)}{\text{argmin}}\sum {\left[i_{q,f}-{\hat{i}}_{q,f}{|}_{\left(\bar{p},\bar{G}\right)}\right]}^{2}$. As mentioned previously, the faulty phase can be determined from ***p***, and *i_q,f_* is equivalent to *i_q_* − *i_q,n_*. Therefore, the proposed method can estimate the faulty phase and *G* if the *i_q,n_* estimation is accurate. This subsection validates the open-loop observers and the parameter estimation, respectively. In the next experiment, the proposed method will be verified.

### 4.2. Estimation Results of Proposed Method

Two experiments were conducted for the verification. One experiment used the accurate parameters: $L_{q}^{\prime }=5.47$ mH, $L_{d}^{\prime }=3.09$ mH, and $\psi_{PM}^{\prime }=0.0976$ Wb; the other experiment used the inaccurate parameters: $L_{q}^{\prime }=7.00$ mH, $L_{d}^{\prime }=4.00$ mH, and $\psi_{PM}^{\prime }=0.1074$ Wb.

**Figure 8:**
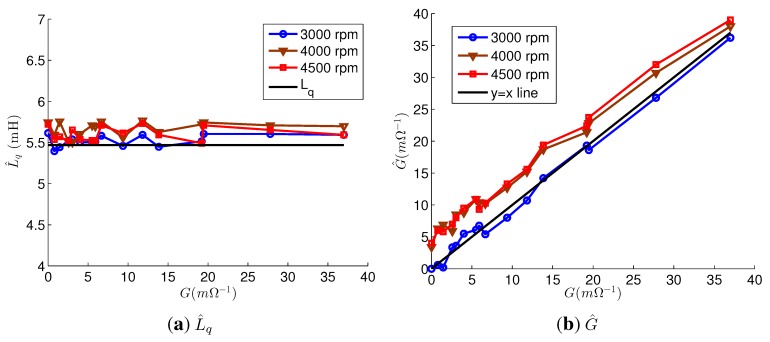
Estimation results of parameter estimation using accurate parameters. (**a**) ${\hat{L}}_{q}$; (**b**) $\hat{G}$.

Figure 8 depicts the estimation results of the parameter estimation using the accurate parameters. The *L_q_* estimations were accurate for every fault; their error rates were below 5.4%. $\hat{G}$ at 3000 rpm was almost equal to *G*; however, the values of $\hat{G}$ at 4000 and 4500 rpm were about 4 mΩ^−1^ higher than those of *G*. Regardless of the speed and *G*, $\hat{p}={\left[0\;0\;1\right]}^{T}$ for the faulty motor. From $\hat{p}$, the faulty phases were correctly estimated as phase *c*. For the healthy motor, $\hat{p}={\left[1\;0\;0\right]}^{T}$ at 3000 rpm and $\hat{p}={\left[0\;0\;1\right]}^{T}$ at 4000 rpm and 4500 rpm. That is, *L_q_* and the faulty phase were correctly estimated, but *G* estimations at 4000 rpm and 4500 rpm had errors even though the accurate parameters were used. The errors were relatively small compared to the values of *G* for the severe fault, and the estimation results were reliable as the values of $\hat{G}$ were high. On the other hand, the estimation results for the mild fault were not reliable. The errors were caused by the difference between ${\hat{i}}_{q,n}$ and *i_q_* of the healthy motor in Figure 6a. This indicates that the estimation error, which is caused by the model uncertainty, makes it difficult to distinguish between a slightly faulty PMSM and a healthy PMSM.

**Figure 9:**
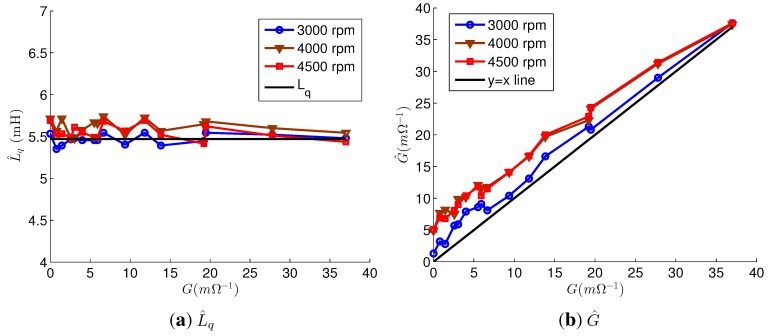
Estimation results of parameter estimation using inaccurate parameters. (**a**) ${\hat{L}}_{q}$; (**b**) $\hat{G}$.

**Figure 10:**
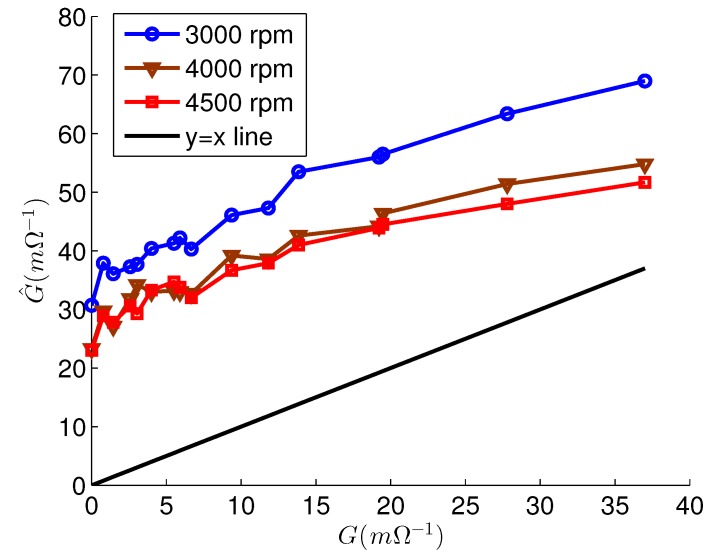
The estimation result $\hat{G}$ without *L_q_* estimation.

Figure 9 shows the estimation results using the inaccurate parameters. The faulty phases were correctly estimated in all experiments for the faulty motor. For the healthy motor, the estimated faulty phases were phase *c* at every speeds. *L_q_* was accurately estimated in every experiments, and their error rates were below 5.0%. As a result, the estimation errors due to Δ*L_q_* were compensated, and $\hat{G}$ values were only slightly increased compared to the $\hat{G}$ values in Figure 8b, because of Δ*L_d_* and Δ*ψ_PM_*. Therefore, $\hat{G}$ was still reliable when the $\hat{G}$ values were high. On the other hand, if *L_q_* was not estimated, $\hat{G}$ exhibited significant errors, and the estimated values were very inaccurate, as shown in Figure 10.

## 5. Conclusions

This paper proposes a diagnostic method for a multipole PMSM under an interturn short-circuit fault. The main objective of the proposed method is to estimate the faulty phase and the value of *G*, which is an index of the severity of the fault. For this reason, two open-loop observers and an optimization method based on a particle-swarm are implemented. For robustness against parameter uncertainties, *L_q_* is also estimated, and high-speed operation of the PMSM is required. In the experiment, the *L_q_* values were accurately estimated and the faulty phases were detected in every fault. The estimated values of *G* had errors, but the high values were reliable. On the other hand, the low values were unreliable and it is difficult to distinguish between a slightly faulty PMSM and a healthy PMSM. This problem is unavoidable in fault diagnosis, but further study is needed to improve the reliability. The experimental results confirmed that the proposed method was robust against parameter uncertainties.
